# Design of Interface ASIC with Power-Saving Switches for Capacitive Accelerometers

**DOI:** 10.3390/mi16010096

**Published:** 2025-01-15

**Authors:** Juncheng Cai, Yongbin Cai, Xiangyu Li, Shanshan Wang, Xiaowei Zhang, Xinpeng Di, Pengjun Wang

**Affiliations:** 1 Hangzhou Sotry Automatic Control Tech Co., Ltd., Hangzhou 311112, China; sotrycjc@163.com (J.C.); yongbincai@sotry.cn (Y.C.); 2 College of Electrical and Electronic Engineering, Wenzhou University, Wenzhou 325035, China; xli@idt.eitech.edu.cn; 3 Ningbo Institute of Digital Twin, Eastern Institute of Technology, Ningbo 315042, China; 4 Faculty of Electrical Engineering and Computer Science, Ningbo University, Ningbo 315211, China; zhangxiaowei@nbu.edu.cn; 5 Shanghai Aerospace Control Technology Institute, Shanghai 201109, China; dixinpeng1@163.com

**Keywords:** MEMS accelerometers, integrated circuits, high-precision

## Abstract

High-precision, low-power MEMS accelerometers are extensively utilized across civilian applications. Closed-loop accelerometers employing switched-capacitor (SC) circuit topologies offer notable advantages, including low power consumption, high signal-to-noise ratio (SNR), and excellent linearity. Addressing the critical demand for high-precision, low-power MEMS accelerometers in modern geophones, this work focuses on the design and implementation of closed-loop interface ASICs (Application-Specific Integrated Circuits). The proposed interface circuit, based on switched-capacitor modulation technology, incorporates a low-noise charge amplifier, sample-and-hold circuit, integrator, and clock divider circuit. To minimize average power consumption, a switched operational amplifier (op-amp) technique is adopted, which temporarily disconnects idle op-amps from the power supply. Additionally, a class-AB output stage is employed to enhance the dynamic range of the circuit. The design was realized using a standard 0.35 μm CMOS process, culminating in the completion of layout design and small-scale engineering fabrication. The performance of the MEMS accelerometers was evaluated under a 3.3 V power supply, achieving a power consumption of 3.3 mW, an accelerometer noise density below 1 μg/√Hz, a sensitivity of 1.65 V/g, a measurement range of ±1 g, a nonlinearity of 0.15%, a bandwidth of 300 Hz, and a bias stability of approximately 36 μg. These results demonstrate the efficacy of the proposed design in meeting the stringent requirements of high-precision MEMS accelerometer applications.

## 1. Introduction

MEMS sensors are extensively utilized in civilian applications, occupying a significant market share [1,2,3,4,5,6,7]. The design and development of high-precision, miniaturized, and integrated accelerometers have become a focal point of global MEMS technology research [8,9,10,11]. With advancements in integrated circuit processes, integrated accelerometers have gained the potential for low-cost, high-volume manufacturing. This trend necessitates the development of accelerometers that align with the consumer electronics market’s demand for miniaturization, low power consumption, and high precision [12,13,14]. A key research direction lies in achieving a wide dynamic range for the sensor element and designing low-power signal detection circuits.

While micro-accelerometers with open-loop output are relatively simple to design, their signal bandwidth is constrained by the sensor element, and their input range is significantly limited. To achieve improved linearity, dynamic range, and signal bandwidth, a closed-loop interface circuit is indispensable. Such circuits can expand the accelerometer’s range without increasing the size of the sensor element [7,15,16]. However, closed-loop detection requires an electrostatic feedback force generated by the interface circuit to drive the sensor element, which significantly increases power consumption compared to open-loop accelerometers. Therefore, optimizing power consumption in closed-loop accelerometers is crucial to improving their overall performance. In response to these challenges, this work proposes an ASIC-based signal detection circuit for capacitive closed-loop accelerometers, addressing both system-level requirements and circuit-level design considerations to achieve a balance between high performance and low power consumption.

### 1.1. Motivation

Small, integrated accelerometers are extensively utilized in high-precision positioning and navigation for micro-nano satellites and vibration detection in the Internet of Vehicles. Accelerometers equipped with readout circuits typically operate in one of two states: open-loop or closed-loop. While open-loop accelerometers offer advantages such as a simple structure, ease of design, and low power consumption, they suffer from limitations in terms of poor linearity and restricted dynamic range within the sensor system. Closed-loop accelerometers, on the other hand, address these limitations but require additional power for the force-feedback mechanism, which includes electrostatic force generation and compensation circuits to drive and stabilize the proof mass. Oscillating or resonant readout circuits further increase power consumption due to the operational requirements of modules such as trans-impedance amplifiers and multipliers. As a result, closed-loop operation is generally preferred for high-precision applications, despite its higher power demands.

### 1.2. Related Work

Achieving sub-μg equivalent resolution in closed-loop accelerometers remains a significant challenge. In [17], the implementation of high-order noise shaping and multi-bit quantizer feedback was demonstrated, albeit only through simulation studies. In [18,19], a novel charge detection method based on a continuous-time (C-T) phase-sensitive modulation and demodulation technique was proposed. Experimental results indicated that this approach could enable the interface circuit to achieve high-resolution outputs. However, the performance of the charge-sensing amplifier is significantly affected by parasitic capacitance and the cascaded low-pass filter in the back-end circuit. These factors introduce noise aliasing and elevate power consumption due to the inherent low-frequency characteristics of the system. Given the narrow bandwidth of MEMS accelerometers, noise shaping can be effectively achieved using a high oversampling ratio (OSR), enabling moderate-speed operation with low power consumption [10]. This highlights the potential for optimized interface circuit designs that balance high resolution and energy efficiency in MEMS accelerometer systems.

In this study, we address the challenge of power consumption in accelerometer interface circuits by proposing a novel power-saving method based on switched-capacitor technology. This approach introduces power-saving switches into the system without altering the original amplifier structure, allowing the amplifier to temporarily enter a dormant state when not in use. By implementing this method, the power consumption of the operational amplifier can be reduced by 30–40% when the duty cycle of the power-saving switches is set to 50%. The power savings become even more significant with longer switch duty cycles in practical applications. To validate the proposed design, an application-specific integrated circuit (ASIC) chip was developed and integrated with vacuum-packaged sensors from Colibrys for testing. Experimental results demonstrate that the integrated accelerometer achieves low power consumption and low noise performance, making it suitable for high-precision applications requiring energy efficiency.

## 2. Interface Circuit

**Figure 1:**
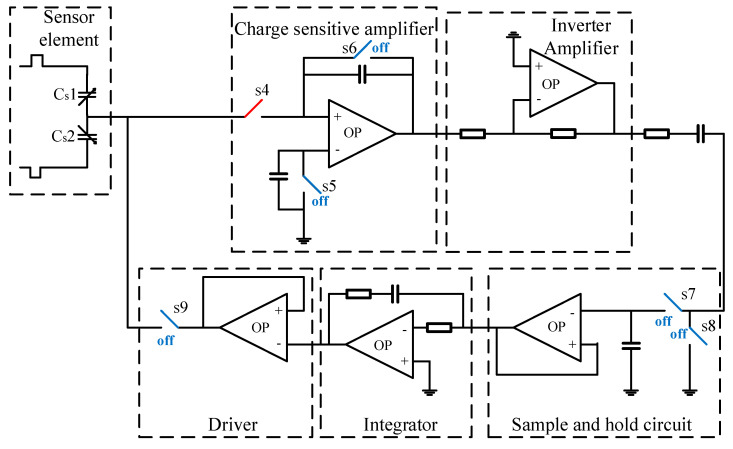
Overall circuit structure.

The interface circuit consists of a charge-sensitive amplifier, inverter amplifier, sample-and-hold circuit, integrator, and driver, as illustrated in Figure 1. In this topology, a timing circuit generates an excitation signal, which is applied to the fixed electrode of the acceleration sensor to produce an electrical signal. This signal is amplified by the preamplifier and processed by the demodulator, resulting in a low-frequency electrical signal that reflects the acceleration. The output analog electrical signal Vout is filtered to meet the required specifications for signal output. The self-test circuit operates by utilizing a pair of differential capacitors as acceleration sensors. Pulse voltage signals with equal amplitude and opposite phases are applied to the fixed electrodes of the capacitors. Due to the common-mode suppression effect at the negative input of the charge amplifier, the potential of the positive input is clamped to zero. When there is a mismatch between the upper and lower capacitors, a charge difference is generated by the pulse voltage signals acting on the differential capacitor pair. This charge difference is then transferred to the charge-sensitive amplifier, producing a voltage proportional to the capacitance difference between the differential capacitors. The amplified signal passes through a series capacitor and a sampling capacitor via a switch. When the switch is closed, the signal is applied to both capacitors; when the switch is open, the signal is sampled by the sampling capacitor. The sampled signal is then further amplified, phase-shifted by the integrator, and processed through a low-pass filter to generate an analog output signal. We have carefully considered the phase shift introduced by each stage, including the charge sensitive amplifier, integrator, and sample-and-hold circuits, during the design process. To minimize its impact, the operational amplifiers were designed with sufficient bandwidth and phase margin to ensure stable operation across the target frequency range. Additionally, the switched-capacitor circuits were analyzed to ensure that their sampling and switching operations did not introduce significant phase delay. Based on our analysis and experimental results, the phase shift introduced by the circuit is negligible within the bandwidth of interest (300 Hz) and does not adversely affect the accelerometer’s performance, including its sensitivity, noise density, or linearity. Simultaneously, the output signal is applied to the middle plate of the differential capacitor pair, generating an electrostatic feedback force that facilitates closed-loop operation of the accelerometer. To ensure adequate signal processing capability in the final stage, the weak driving capability of the resulting voltage is enhanced using a buffer amplifier. This provides sufficient drive strength for downstream signal processing, ensuring the robustness of the closed-loop accelerometer system.

### Pre-Stage Low-Noise Charge Amplifier

As the acceleration signal changes, the displacement of the mass block of the sensor element causes a change in capacitance because of the distance or area between the substrate and the sensor element. In this way, the signal of acceleration can be obtained by detecting the change in capacitance. To increase the detection capacitance area, the common structure of the sensor element is the multi-finger capacitor. In this work, we applied the VS9010 from Colibrys company (Yverdon-les-Bains, Switzerland). Its open-loop resonant frequency is 1 kHz, the quality factor of sensor structure is greater than 30, and the corresponding Brownian noise is less than 25 μV/√Hz. The static capacitance of the sensor structure is 180 pF, and the sensitivity is 10 pF/g. The main parameters are shown in Table 1.

The integrated accelerometer operates at low power consumption, making the folded-cascade structure particularly appealing due to its ability to achieve high impedance and high gain by connecting transistors in series. However, this approach comes at the cost of a reduced dynamic range. In contrast, the multi-stage amplifier design features only two transistors in the output stage, which satisfies the output swing requirements and is generally more suitable for low-voltage processes. It should be noted that the output dynamic range of the circuit decreases by over 15% with each additional transistor connected in series within the output stage.

To meet accuracy requirements, the operational amplifier (op-amp) must achieve a DC gain of at least 60 dB. Furthermore, a phase margin of no less than 45° is essential to provide adequate tolerance for process variations. In switching op-amp configurations, a phase margin of 45° is also necessary to ensure critical damping when responding to step input signals. The unity-gain frequency of the op-amp plays a pivotal role in determining the settling time and ensuring the accuracy of the filter’s transfer function, as it is directly linked to the gain-bandwidth product. Generally, a higher unity-gain frequency results in shorter settling times and improved filter performance. However, an excessively high unity-gain frequency can lead to significant noise aliasing in switched-capacitor (SC) circuits and increased power consumption. To strike a balance between minimizing noise aliasing and maintaining the integrity of the filter’s transfer function, the unity-gain frequency is typically set to approximately 10 times the sampling frequency of the target SC circuit.

The duty cycle of the sample switching clock is 50%; the time from the switch on to the final value of the output voltage must be less than half a clock cycle. The above condition can be satisfied only if the switching time *T_switching_on_*, swing time *T_swing_*, and build-up time *T_settle_* of the op-amp are less than half a clock cycle.

(1) $$T_{switching\_on}+T_{slew}+T_{settle}<\frac{T}{2}=\frac{1}{2f_{s}}$$

Due to the requirements for turn-on time, switching operational amplifiers (op-amps) operate at slower speeds compared to conventional op-amps under the same specified current. When operating at moderate frequencies (below 1 MHz), it is reasonable to allocate approximately 20% of the half-clock cycle for the op-amp’s turn-on time. However, at higher frequencies (ranging from several MHz to tens of MHz), the turn-on time of a switching op-amp can account for 40% to 50% of the half-clock cycle. This extended turn-on time is typically determined by the specific turn-on mechanism of the switching op-amp.

The settling time can be as much as 40% of the half-clock cycle with an accuracy requirement of 0.1%. The settling time is governed by the −3 dB bandwidth, which is in turn influenced by the unity-gain frequency and the closed-loop gain. For folded-cascade op-amps, the unity-gain frequency is determined by the load capacitance. Consequently, the settling time is also dependent on the equivalent load capacitance, especially in switched-capacitor (SC) circuits where capacitive feedback is commonly present. For secondary-stage op-amps, the unity-gain frequency is primarily determined by the compensation capacitance. The swing time of the op-amp is determined by the swing rate of the op-amp and the change of the voltage Δ*V_out_* at the output.

(2) $$\Delta V_{out}=V_{out}(nT)-V_{out}((n-1)T)$$

(3) $$T_{slew}=\frac{\Delta V_{out}}{SR}$$

The maximum Δ*V_out_* occurs when it is processing the largest passband frequencies. In the worst case, *V_out(t)_* is sampling a continuous time signal *V(t)* with frequency **ω***_B_* and amplitude *V_swing_*. Thus, the maximum step of *V_out(t)_* occurs at *T* = 1/*f_s_*, which is given as follows:

(4) $$V(t)=V_{swing}\sin\omega_{B}t$$

(5) $$\Delta V_{out,\max}=T\left|\frac{dV(t)}{dt}\right|\max=\frac{\omega_{B}V_{swing}}{f_{s}}$$

where *T* = 1/*f_s_*, *p* is the proportion of the half-cycle allowed for the swing time, the requirement for the swing rate can be given by the following equation:

(6) $$SR\geq \frac{V_{out,\max}}{T_{slew}}\approx \frac{\omega_{B}V_{swing}}{sf_{s}(T/2)}=\frac{2\omega_{B}V_{swing}}{x}$$

In this equation, *T_slew_* represents the allowable slew time, which is the time required for the output voltage of the operational amplifier to reach its target level during a sampling period. The term *s* is a dimensionless proportionality factor that indicates the fraction of the half-period *T*/2 allocated for the slew time. For instance, if *s =* 0.5, the slew time occupies half of the half-period *T*/2. This factor is critical for ensuring that the operational amplifier has sufficient time to settle without introducing significant errors. The parameter x is a design margin factor for the required slew rate (SR). It accounts for practical considerations in switched-capacitor (SC) circuit design and provides a buffer to ensure that the operational amplifier can achieve the necessary output swing within the allocated slew time. In most switched-capacitor (SC) circuits, the typical value of the parameter x is approximately 40%. A fully differential architecture is commonly employed to improve performance by reducing common-mode rejection ratio (CMRR), power supply rejection ratio (PSRR), and noise. In a two-stage operational amplifier (op-amp), the first stage with high gain is critical for optimizing noise performance. In a differential topology, the common-mode gain can ideally be reduced to zero. However, at low frequencies, power supply noise can couple to the output due to bias current variations and mismatches in the differential transistor pair. At high frequencies, to mitigate the impact of parasitic capacitances, it is essential to minimize their effects in the circuit layout. This is typically achieved by maximizing the input and feedback capacitance in the layout design, which helps to suppress the influence of parasitic capacitances on circuit performance.

**Figure 2:**
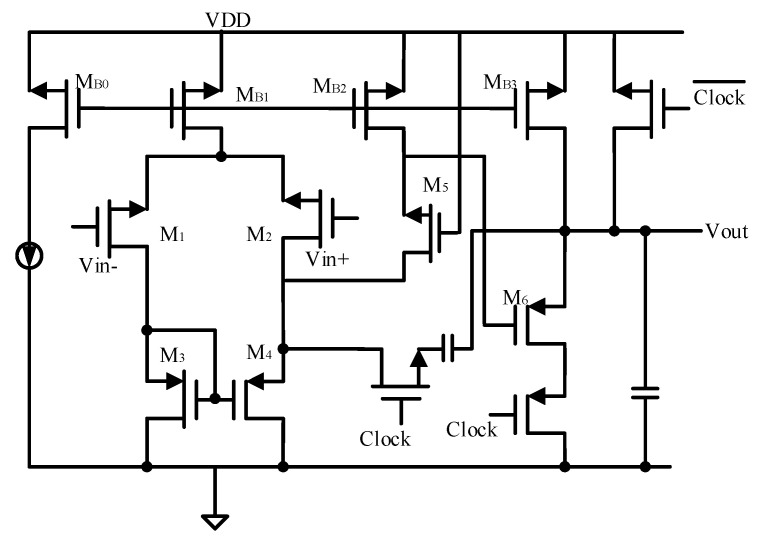
Switched-op-amp with switched output stage.

**Figure 3:**
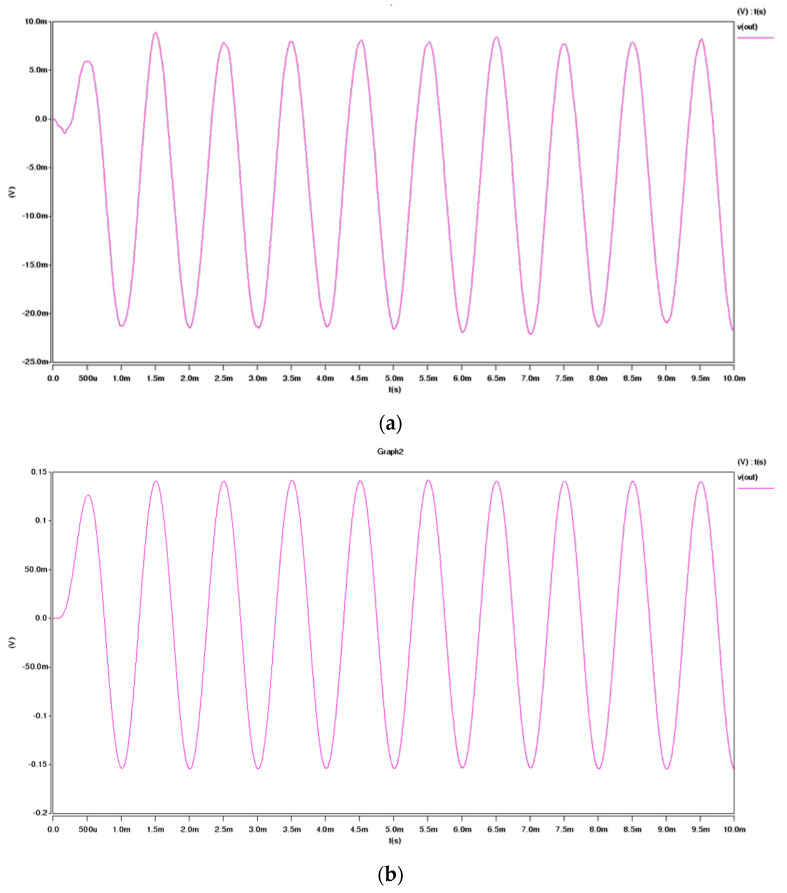
System schematic simulation results. (**a**) Detection results when the acceleration signal is 1 g. (**b**) Detection results when the acceleration signal is 100 mg.

Since the drive current for the load is supplied by the output stage of the op-amp, the output stage typically accounts for a significant portion of the overall current consumption of the op-amp. To achieve a reduction in power consumption, it is feasible to selectively turn off only the output stage. Figure 2 illustrates the design of a switching op-amp featuring a switchable output stage. As shown in Figure 2, the switching speed is primarily determined by the turn-on time of the current source transistor MB3. To minimize the time required to recharge the compensation capacitor, a switch (MSW) is incorporated into the compensation feedback circuit. This design ensures that the compensation capacitor remains undischarged when the output stage is off and avoids additional charging when the output stage is on. Furthermore, during normal operation, the switch MSW functions as a zeroing resistor for the second-stage op-amp, effectively eliminating the right-half-plane zero. However, this design necessitates additional biasing for MSW, which increases overall power consumption. Figure 3 presents the simulation results of the closed-loop system. The results demonstrate that the proposed system architecture exhibits excellent signal detection capabilities, highlighting its effectiveness in achieving the desired performance.

**Figure 4:**
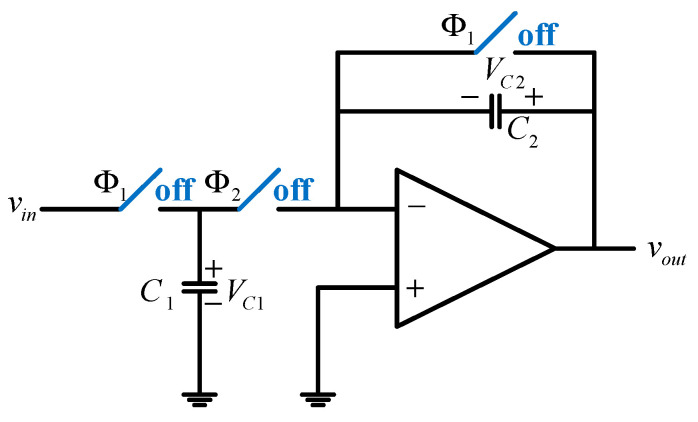
Switched-op-amp topology.

**Figure 5:**
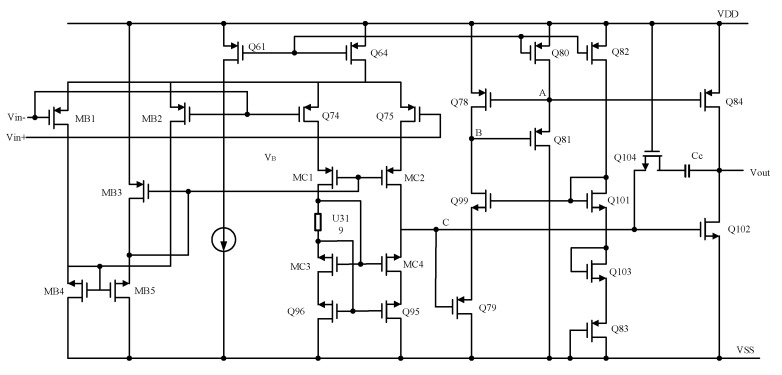
The transistor circuit in switched-op-amp.

In the design of the interface circuit, a switched-capacitor configuration was employed to integrate power-saving switches without altering the original amplifier architecture, as shown in Figure 4. The transistor circuit in switched-op-amp is shown in Figure 5. This approach allows the amplifier to enter a temporary dormant state when it is not actively processing signals. By implementing this method, the power consumption of the operational amplifier can be reduced by 30% to 40% when the switch operates with a 50% duty cycle. Furthermore, the power savings become increasingly significant as the duty cycle of the switch is extended during actual operation.

**Figure 6:**
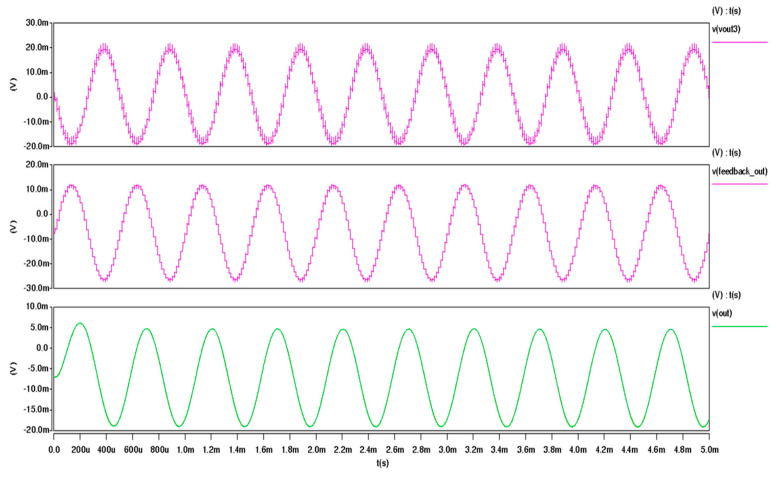
Simulation result of the whole circuit.

The output signal v(vout3) represents the signal obtained prior to sampling. As observed, the signal waveform exhibits numerous spikes caused by the switching effect; however, these do not impact the final measurement result. The switching effect is eliminated after the sampling process, and subsequent processing by the integrator and feedback driver circuit produces the signal v(feedback_out). This output, which is applied to the feedback beam, is a step-like signal that is directly proportional to the acceleration value. The integrator, responsible for generating the required phase shift, incorporates resistors and capacitors that can be adjusted off-chip to meet specific system requirements. Ultimately, a continuous analog measurement signal is obtained through a low-pass filter. To maintain the general applicability of the chip, no active filtering components are integrated within the chip itself. Instead, external filtering can be implemented as needed to achieve optimal performance for a given application. The simulation result of the whole circuit is as shown in Figure 6.

## 3. Test Results and Analysis

### 3.1. Micro Accelerometer System Testing

**Figure 7:**
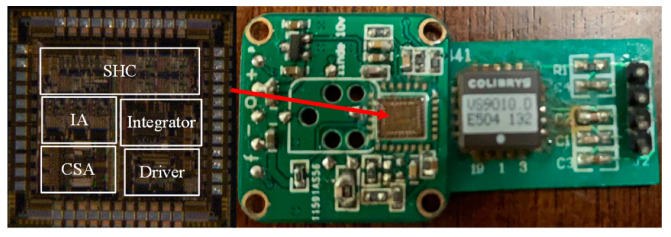
Chip photos and MEMS accelerometer test board.

Following comprehensive system analysis, circuit parameter optimization, circuit simulation refinement, and layout design, the MEMS accelerometer interface circuit described in this paper was successfully implemented. Using Cadence simulation tools and a 0.35 μm four-layer metal polysilicon process, the layout design and post-simulation validation were completed. The engineering batch production was carried out utilizing the standard 0.35 μm CMOS process provided by Shanghai Hua Hong. The fabricated chip and the corresponding accelerometer test board are shown in Figure 7. The chip occupies an effective area of 7.8 mm^2^ and includes a total of 45 PAD points. Beyond the power supply PADs and input/output PADs, the majority of the remaining PADs serve as test ports for individual modules. To comprehensively evaluate the accelerometer’s performance, a dedicated test board was designed to facilitate detailed static and dynamic performance measurements. The sensor system was powered using an Agilent E3631 power supply with an adjustable voltage range of 3.3 V to 5 V. While reducing the supply voltage decreases the sensor’s overall power consumption, a higher supply voltage improves the noise performance of the front-end charge conversion circuit and enhances the sensor’s output accuracy. During dynamic performance testing, the sensor was operated at a 3.3 V supply voltage, corresponding to reference voltages of 1.65 V. The sensor’s sampling period was set to 4 μs, and a 2 MHz square wave generated by an external crystal oscillator served as the clock source. The timing for the control switches was generated internally by the chip’s clock divider circuit.

**Figure 8:**
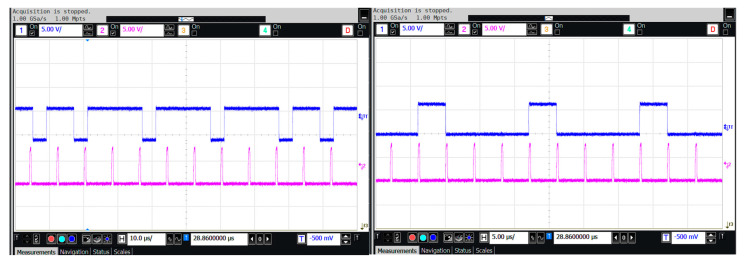
The transient test for micro-accelerometers.

For the static functionality test, the self-test input port was set to an intermediate reference level. The accelerometer was subjected to DC accelerations of +1 g and −1 g, respectively, and the output was observed using an oscilloscope to verify the sensor’s proper operation. The output bitstream waveforms of the MEMS accelerometer under the DC acceleration inputs are shown in Figure 8.

**Figure 9:**
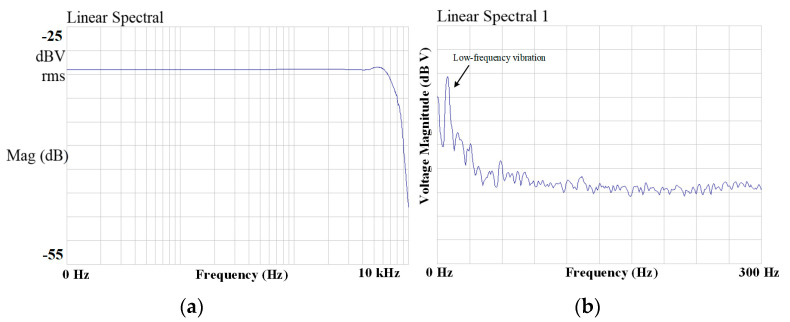
Frequency response and noise spectrum closed-loop micro-accelerometers.

In the oscilloscope waveform, the upper section displays the accelerometer’s output bitstream, while the lower section shows the control clock waveform of the quantizer. When a +1 g DC acceleration is applied, the output bitstream of the sensor predominantly consists of high levels, with a duty cycle significantly exceeding 50%. When −1 g DC acceleration is applied, the output bitstream is primarily low level, with a duty cycle much smaller than 50%. These results confirm the correct functional operation of the accelerometer. The comprehensive test results of the micro-accelerometer interface ASIC chip and the integrated system, which includes the ASIC chip matched with the sensitive structure, are summarized in Table 2. The results demonstrate that the interface circuit supports the micro-accelerometer in achieving a 1 μg output noise density. The frequency response test of the micro-accelerometer is shown in Figure 9, indicating an effective bandwidth exceeding 1 kHz with high in-band flatness. These findings confirm that the switched-capacitor interface ASIC chip, based on the principle of time-division electrostatic feedback, successfully enables the closed-loop operation of the micro-accelerometer with electrostatic force feedback.

Following static and dynamic testing, performance analysis of the accelerometer was conducted. Given the low signal bandwidth of the accelerometer, output noise levels at low frequencies were carefully examined. Noise testing was carried out using an HP35670A dynamic signal analyzer, and the results are presented in Figure 9. To minimize the impact of external electromagnetic interference on the test results, environmental damping and electromagnetic shielding measures were implemented. However, despite these precautions, low-frequency environmental vibration interference remains evident. The resonant frequency of the mechanical sensing structure designed in this study is approximately 1 kHz, resulting in a pronounced noise spike at the resonant frequency. This resonance not only restricts the sensor’s effective application bandwidth but also amplifies noise near the resonant point, thereby degrading the overall performance of the sensor.

**Figure 10:**
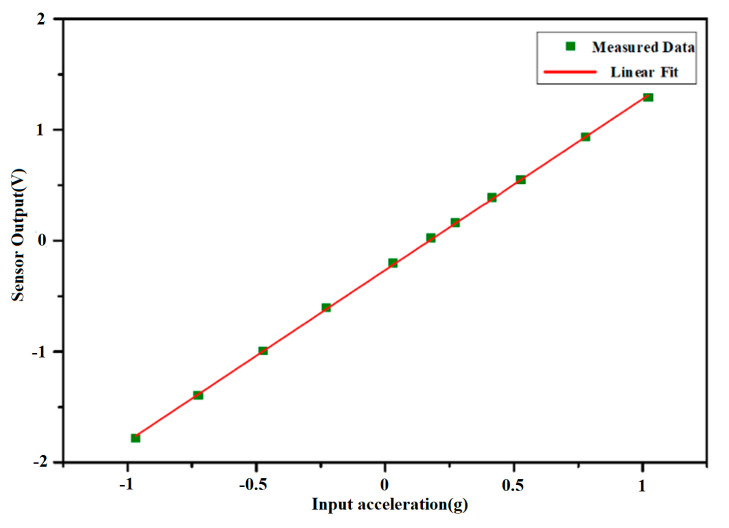
The linearity test.

**Figure 11:**
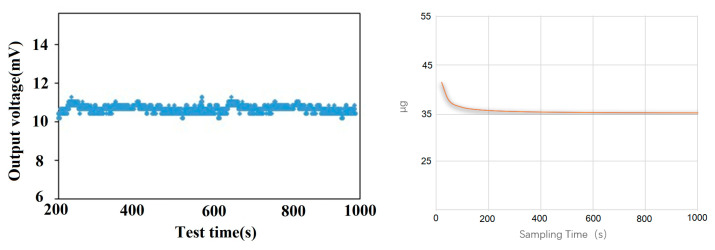
The bias instability test.

To evaluate sensor linearity, a high-precision vibration table was employed. Linearity testing involved sampling 11 data points between −1 g and +1 g, including a zero-point measurement at 0 g, with intervals of 0.16 g between each subsequent point. The test results indicate a nonlinearity of 0.15% FS as shown in Figure 10. The overall system’s nonlinearity is primarily constrained by the structural design of the accelerometer sensor itself. For bias stability testing, the accelerometer output was sampled under zero-acceleration input in a controlled laboratory environment. Sampling was performed over a duration exceeding four hours. The sampled data were processed and analyzed using a Matlab (R2016a, MathWorks, Natick, MA, USA) Allan variance program to derive the bias stability of the closed-loop micro-accelerometer. The calculated bias stability was approximately 36 μg, as shown in Figure 11. This performance is further improved with enhanced electromagnetic shielding and vibration damping techniques.

### 3.2. Micro Accelerometer Performance Comparison

The performance metrics of the ΣΔ micro-accelerometer developed in this study were compared against advanced performance benchmarks reported in the international literature. The comparison was carried out using the representative Figure of Merit (FOM), calculated based on the following expression:

(7) $$FOM=\frac{Pa_{n}\sqrt{BW}}{BW}$$

where *P* represents the total power consumption of the chip, *a_n_* denotes the equivalent input acceleration noise, and *BW* is the signal bandwidth. The *FOM* value serves as a comprehensive indicator of the overall performance of the sensor.

The results of this comparison demonstrate that the accelerometer presented in this work achieves competitive performance. Compared to the power consumption reported in [20,21,22,23], this work is the best. The *FOM* value in this work exceeds those of the design proposed in reported papers. Following parameter optimization, the noise floor is primarily determined by the performance of the front-end charge amplifier. Considering power consumption, harmonic distortion, and noise performance, the interface ASIC for micro-accelerometers developed in this study achieves superior performance. These results indicate that the accelerometer is well-suited for high-precision applications.

## 4. Conclusions

In this study, an interface ASIC micro-accelerometer interface circuit was designed and implemented, achieving excellent overall performance suitable for high-precision applications. Through comprehensive system analysis, circuit optimization, and experimental verification, the proposed design demonstrated low equivalent input acceleration noise, low power consumption, and wide signal bandwidth. The use of a high-Q vacuum-packaged mechanical structure effectively minimized thermal noise, while noise shaping within the signal bandwidth further suppressed quantization noise. The noise floor was primarily influenced by the performance of the front-end charge amplifier, which was optimized to achieve superior results. Compared to advanced designs reported in the literature, the accelerometer developed in this work achieves a competitive FOM value, balancing power consumption, noise performance, and harmonic distortion. These outcomes validate the suitability of the proposed design for demanding high-precision applications in various fields.

## Figures and Tables

**Table 1:** Parameters of the sensor element.

Parameters	Value
Sensitivity	200 ± 2 mV/g
Full scale range	±10 g
Bandwidth (−3 dB)	0 to ≥2.4 kHz
Resonant frequency (ω0)	2 kHz
Nonlinearity	<1% FS
Noise spectral density in band	25 μV/√Hz

**Table 2:** Comparison of the performance of sigma-delta in this work and those reported works.

	Process	Supply/Range	Power	Bandwidth	Noise Floor	FOM
[20]	0.35 μm	3.6 V/±1.15 g	3.6 mW	200 Hz	2 μg/√Hz	0.51
[21]	0.7 μm	5 V/12 V/±1.5 g	85.8 mW	300 Hz	0.3 μg/√Hz	1.49
[22]	0.5 μm	3 V/NA	4.5 mW	500 Hz	4 μg/√Hz	0.80
[23]	0.35 μm	5 V/±1.2 g	7.7 mW	200 Hz	0.5 μg/√Hz	0.27
This work	0.35 μm	3.3 V/±1 g	3.3 mW	300 Hz	1 μg/√Hz	0.19

## Data Availability

The original contributions presented in this study are included in the article. Further inquiries can be directed to the corresponding authors.
